# Lifespan Extension Conferred by Endoplasmic Reticulum Secretory Pathway Deficiency Requires Induction of the Unfolded Protein Response

**DOI:** 10.1371/journal.pgen.1004019

**Published:** 2014-01-02

**Authors:** Vyacheslav M. Labunskyy, Maxim V. Gerashchenko, Joe R. Delaney, Alaattin Kaya, Brian K. Kennedy, Matt Kaeberlein, Vadim N. Gladyshev

**Affiliations:** 1 Division of Genetics, Department of Medicine, Brigham and Women's Hospital and Harvard Medical School, Boston, Massachusetts, United States of America; 2 Department of Pathology, University of Washington, Seattle, Washington, United States of America; 3 Department of Biochemistry, University of Washington, Seattle, Washington, United States of America; 4 Buck Institute for Research on Aging, Novato, California, United States of America; Stanford University Medical Center, United States of America

## Abstract

Cells respond to accumulation of misfolded proteins in the endoplasmic reticulum (ER) by activating the unfolded protein response (UPR) signaling pathway. The UPR restores ER homeostasis by degrading misfolded proteins, inhibiting translation, and increasing expression of chaperones that enhance ER protein folding capacity. Although ER stress and protein aggregation have been implicated in aging, the role of UPR signaling in regulating lifespan remains unknown. Here we show that deletion of several UPR target genes significantly increases replicative lifespan in yeast. This extended lifespan depends on a functional ER stress sensor protein, Ire1p, and is associated with constitutive activation of upstream UPR signaling. We applied ribosome profiling coupled with next generation sequencing to quantitatively examine translational changes associated with increased UPR activity and identified a set of stress response factors up-regulated in the long-lived mutants. Besides known UPR targets, we uncovered up-regulation of components of the cell wall and genes involved in cell wall biogenesis that confer resistance to multiple stresses. These findings demonstrate that the UPR is an important determinant of lifespan that governs ER stress and identify a signaling network that couples stress resistance to longevity.

## Author Summary

Impaired protein function caused by protein misfolding and aggregation has been implicated in the development of age-related diseases and regulation of lifespan. Accumulation of misfolded proteins in the endoplasmic reticulum, a cellular organelle responsible for protein folding and trafficking, activates protective signaling pathways that restore protein homeostasis. One such conserved signalling pathway is mediated by the protein misfolding sensor Ire1p and the transcription factor Hac1p, which up-regulate endoplasmic reticulum chaperones, oxidative folding components and factors that facilitate degradation of misfolded proteins to alleviate increased protein folding demand. Here, we describe the role of the Ire1p pathway and its downstream targets in regulation of lifespan in yeast. While the loss of Ire1p itself had little effect on lifespan, we found that selective inactivation of the individual protein folding and maturation factors led to increased longevity. We also provide evidence that this increased longevity depends on functional Ire1p and induction of multiple cytoprotective pathways that confer resistance to stress.

## Introduction

Membrane and secretory proteins fold into their native conformations in the endoplasmic reticulum (ER) assisted by chaperones, thiol-disulfide oxidoreductases and other systems supporting protein post-translational control. Impairments in this complex process cause unfolded proteins to accumulate, provoking ER stress. Adaptation to ER stress is dependent on the unfolded protein response (UPR) signaling pathway that senses accumulation of unfolded proteins in the ER and restores ER homeostasis by (i) temporarily inhibiting protein synthesis, (ii) degrading misfolded or unassembled proteins, and (iii) increasing expression of chaperones and oxidative folding components that facilitate protein folding [1]. However, depending on the severity and timing of ER stress, it may also lead to cell death when adaptive mechanisms fail.

In mammalian cells, the UPR consists of multiple signaling cascades that are activated by three known ER stress sensor proteins, inositol-requiring protein 1 (IRE1), activating transcription factor 6 (ATF6), and double-stranded RNA-activated protein kinase-like ER kinase (PERK) [2]–[4]. Among these signal transducers, only IRE1 is conserved in budding yeast and is solely responsible for the UPR activation in *Saccharomyces cerevisiae* [5], [6]. Ire1p is an ER-localized transmembrane protein, containing kinase and endoribonuclease (endo-RNase) enzyme activities. Upon activation by ER stress, Ire1p undergoes oligomerization and autophosphorylation [7], [8]. In turn, Ire1p autophosphorylation activates its endo-RNAse domain, which facilitates the excision of an intron and unconventional splicing of *HAC1* mRNA in yeast [9]. Spliced *HAC1* mRNA codes for a functional transcription factor capable of inducing transcription of genes that enhance ER protein folding capacity and alleviating ER stress [10]. However, Ire1p may have other, Hac1p transcription factor-independent functions. In addition to up-regulation of the UPR target genes, metazoan IRE1 has been implicated in degradation of ER-localized mRNAs through its endonuclease activity [11]–[13]. Such ER-localized mRNA decay occurs during prolonged irremediable ER stress due to higher-order oligomerization and hyperactivation of IRE1 [12]. Thus, IRE1 may play a dual role in cell fate, by both allowing cellular adaptation to increased protein folding load and promoting apoptosis depending on the severity of ER stress.

ER stress and protein misfolding are increasingly recognized as contributing factors to the pathophysiology of age-related diseases and aging [14]. Moreover, studies involving model organisms demonstrate that improved ER stress resistance is often associated with increased lifespan and healthy aging [15]–[18]. However, the role of UPR signaling and individual components of ER stress response in regulating lifespan is not known. In this study, we investigated the contribution of the UPR and its downstream targets, including chaperones, oxidative folding components and components of the ER-associated degradation (ERAD), to aging and examined the mechanism of such regulation in a simple model organism, *S. cerevisiae*. We demonstrate that modulation of the UPR by genetic means can extend yeast lifespan, and that induction of UPR signaling is required for activation of multiple stress response pathways that drive lifespan extension.

## Results

### Components of the ER stress response pathway differentially modulate replicative lifespan in *S. cerevisiae*

**Figure 1:**
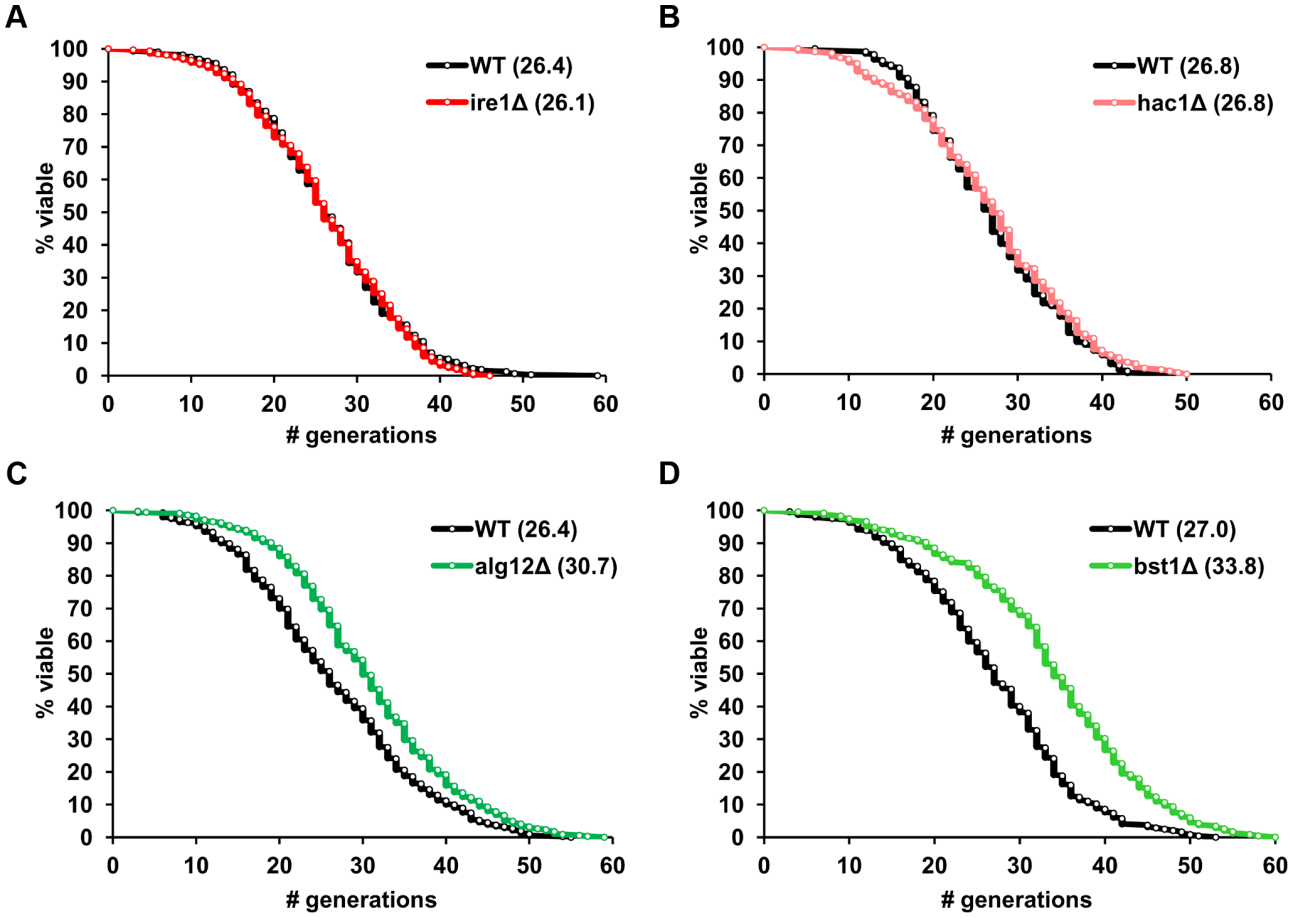
ER stress response genes differentially modulate yeast replicative lifespan. (**A–D**) Survival curves for *ire1Δ*, *hac1Δ*, *alg12Δ* and *bst1Δ* deletion strains. Replicative lifespan data for the strains from both the MATa and MATα ORF deletion collections are pooled, and experiment-matched wild-type cells are shown. Mean lifespans are shown in parentheses.

Analysis of replicative lifespan, which is defined as the number of times each yeast cell divides before it undergoes senescence, is based on the ability of budding yeast to divide asymmetrically producing distinct mother and daughter cells and is often used as a model of aging in mitotically active cells [19]. To examine the relationship between ER stress response genes and aging, we measured replicative lifespan of mutant *S. cerevisiae* strains lacking individual components of the UPR and its transcriptional targets. In addition to *IRE1* and *HAC1*, several downstream effector genes were analyzed including chaperones (*KAR2*), oxidative folding (*ERO1*, *EUG1*, *MPD1*, *PDI1*) and ER-associated degradation (ERAD) components (*DER1*, *SEL1*, *HRD1*), as well as genes involved in N-linked glycosylation (*ALG3*, *ALG12*, *DIE2*, *OST3*, *OST6*) and protein trafficking (*BST1*) [10], [20]. We found that deletion of either *IRE1* or *HAC1*, two genes that are involved in sensing accumulation of unfolded proteins in the ER, did not affect yeast lifespan (**Figure 1A, B**). Unexpectedly, many of the downstream UPR target mutants, 9 out of 14, were found to be significantly long-lived compared to experiment-matched control wild-type cells (**Figure 1C, D** and **Table 1**). Hereafter, we refer to these mutants as “long-lived ER secretory pathway mutants” or “long-lived UPR target gene deletion mutants”. These data demonstrate that components of the ER stress response pathway may differentially modulate replicative lifespan and are important determinants of longevity in *S. cerevisiae*.

**Table 1:** Regulation of lifespan by ER stress response mutants*.

Deletion			Experiment-matched Control			% Mean RLS Change	Ranksum P-Value
Genotype	Mean RLS	N	Genotype	Mean RLS	N		
**UPR**							
*ire1Δ*	26.1	315	WT	26.4	315	−1.1	0.9562
*hac1Δ*	26.8	220	WT	26.8	220	0.0	0.7649
**Oxidoreductases**							
*ero1*-DAmP	28.1	160	WT	22.0	160	28.1	<0.0001
*eug1Δ*	29.8	205	WT	26.8	225	11.2	0.0037
*mpd1Δ*	27.6	115	WT	23.6	115	16.9	0.0206
*pdi1*-DAmP	24.7	70	WT	25.2	70	−2.0	0.6599
**ERAD**							
*hrd1Δ*	29.4	325	WT	24.9	364	18.1	<0.0001
*der1Δ*	29.0	295	WT	24.9	314	16.5	<0.0001
*sel1Δ*	20.0	119	WT	26.3	120	−24.0	<0.0001
**N-linked glycosylation**							
*alg12Δ*	30.7	345	WT	26.4	404	16.3	<0.0001
*alg3Δ*	28.5	240	WT	26.0	240	9.6	0.0027
*die2Δ*	21.3	80	WT	25.6	80	−16.8	0.0032
*ost3Δ*	28.4	75	WT	23.7	75	19.8	0.0058
*ost6Δ*	25.3	80	WT	24.6	80	2.8	0.4617
**Protein trafficking**							
*bst1Δ*	33.8	235	WT	27.0	245	25.2	<0.0001
**Chaperones**							
*kar2*-DAmP	15.5	70	WT	25.2	70	−38.5	<0.0001

* Replicative lifespan data were pooled for strains from both the MATa and MATα ORF deletion collections (See **Table S1** for lifespan data obtained for each mating type separately).

### Lifespan extension in mutants lacking downstream components of the UPR is associated with elevated basal UPR activity

The observation that UPR target gene deletions extend lifespan was counterintuitive, as genes activated by UPR are perceived as protective factors required to restore ER homeostasis, and their deletion might be expected to decrease lifespan. The unexpected and consistent lifespan extension by UPR target gene inactivation may be attributable to hormesis, a phenomenon by which limited stress elicits response mechanisms that protect against similar but higher level stresses associated with aging. To study the molecular mechanisms by which reduced levels of UPR target genes lead to lifespan extension, we focused on two well-characterized genes *ALG12* and *BST1*. Alg12p is an enzyme that catalyzes one of the steps in the synthesis of N-linked glycans [21], whereas Bst1p performs removal of the inositol acyl group required for the quality control of ER to Golgi transport of glycosylphosphatidylinositol-anchored proteins [22].

**Figure 2:**
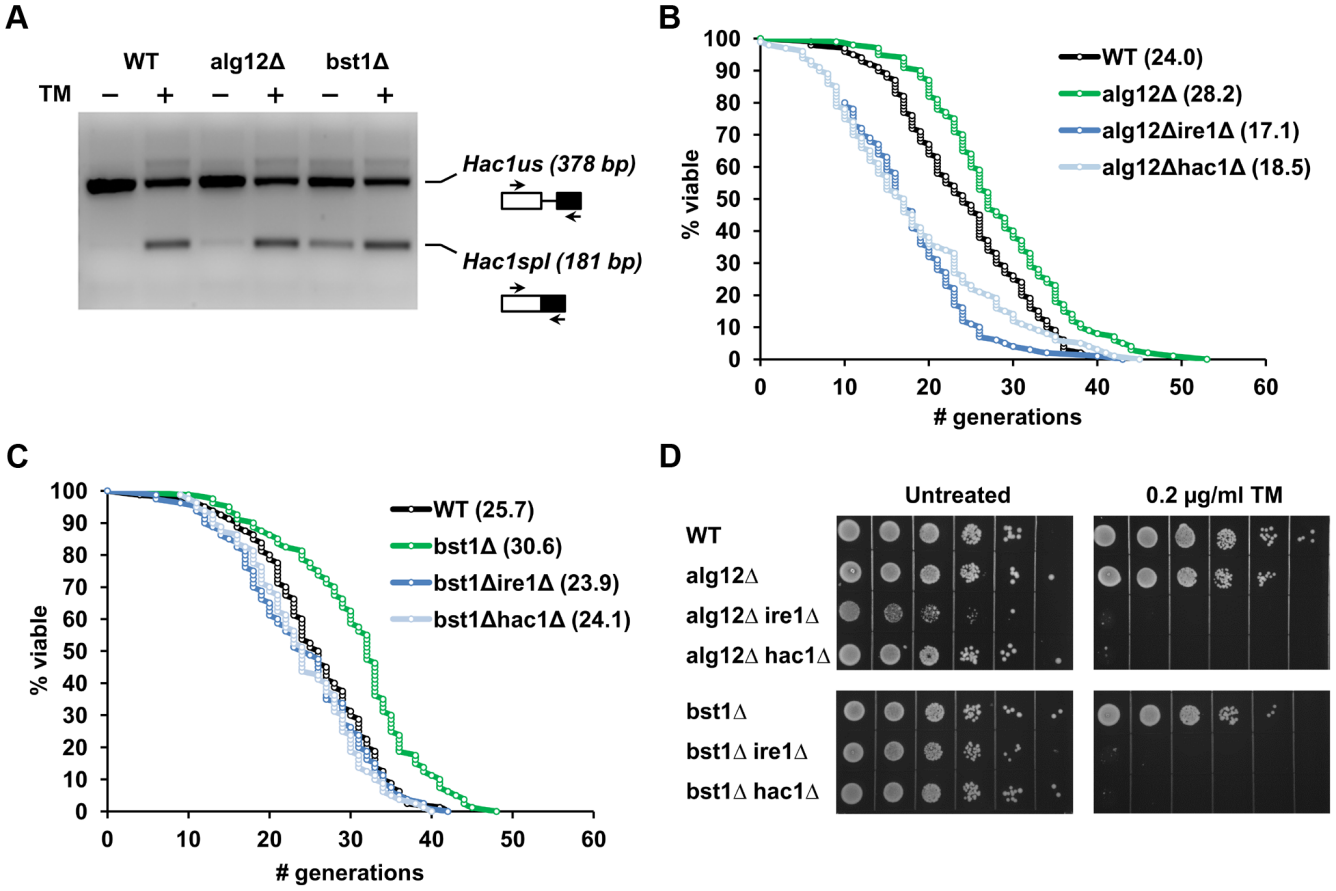
Extended lifespan in *alg12Δ* and *bst1Δ* mutants is dependent on functional Ire1p and Hac1p and is associated with increased basal UPR activity. (**A**) Analysis of *HAC1* mRNA splicing in wild-type, *alg12Δ* and *bst1Δ* cells treated with or without 1 µg/ml tunicamycin for 1 h. Sliced (spl) and unslpliced (us) *HAC1* mRNA were detected by RT-PCR. The image was inverted to negative for better clarity. (**B, C**) Survival curves for *alg12Δ* and *bst1Δ* and the corresponding double mutant strains combining the long-lived gene deletion with either *ire1Δ* or *hac1Δ*. (**D**) Sensitivity of *alg12Δ* and *bst1Δ* and the corresponding double mutant strains to ER stress. For each strain 10× serial dilutions of logarithmically growing cells were spotted on agar plates without the drug (untreated) or plates containing 0.2 µg/ml tunicamycin (TM). Pictures were taken after 48 h incubation at 30°C.

We hypothesized that deletion of genes downstream of the UPR may lead to constitutive activation of Ire1p and induction of UPR dependent cytoprotective pathways. To test this hypothesis, we analyzed whether the level of UPR activity may correlate with the lifespan in *alg12Δ* and *bst1Δ*. Analysis of *HAC1* mRNA splicing was used to monitor the level of UPR activity in wild-type cells and corresponding mutants that were grown in the absence or presence of pharmacological ER stressor tunicamycin for 12 h (**Figure 2A**). In wild-type cells the basal level of UPR activity was very low, as evidenced by the fact that most of the detected *HAC1* mRNA (99%) represented the unspliced form and only 1% corresponded to the spliced form. Treatment of wild-type cells with tunicamycin increased the fraction of spliced *HAC1* mRNA to 31%. In contrast, deficiency of the UPR transcriptional targets, *ALG12* and *BST1*, was associated with increased basal *HAC1* mRNA splicing (7% and 21% of *HAC1* mRNA was spliced for *alg12Δ* and *bst1Δ*, respectively). These data were also in good agreement with ribosome profiling data (see below), which showed different level of *HAC1* translational activation in *alg12Δ* and *bst1Δ* mutants. In addition, we confirmed the level of UPR activation by analyzing the expression of Kar2p, an ER chaperone that is induced by UPR, and found increased Kar2p levels in the long-lived mutants, compared to the mutants that do not affect lifespan (**Figure S1A**). Taken together, our data indicate that lifespan extension conferred by deficiency of UPR components downstream of Ire1p, including *ALG12* and *BST1*, is associated with increased basal UPR activity.

### Deletion of *IRE1* and *HAC1* prevents lifespan extension in UPR target gene deletion mutants

To address whether lifespan extension in strains lacking UPR target genes is dependent on functional Ire1p (an ER stress sensor) and Hac1p (an ER stress-responsive transcription factor), we generated double mutant strains combining the long-lived *alg12Δ* and *bst1Δ* deletions with either *ire1Δ* or *hac1Δ*. If high basal UPR activity is required for increased longevity in the ER secretory pathway mutants, one would predict that deletion of either *IRE1* or *HAC1* should attenuate lifespan extension in these mutants. Consistent with this hypothesis, we observed decreased lifespan in *alg12Δire1Δ* and *alg12Δhac1Δ* double mutants compared to *alg12Δ* (p<0.0001) (**Figure 2B** and **Table S2**). Moreover, both double mutants were significantly shorter-lived than the wild-type strain (p<0.0001). Since a single deletion of either *IRE1* or *HAC1* did not affect yeast replicative lifespan under unstressed conditions (**Figure 1**), these data suggest an adverse genetic interaction of *ALG12* deletion with that of *IRE1* or *HAC1*. We also found that lifespan extension conferred by *bst1Δ* deletion was significantly reduced in *bst1Δire1Δ* and *bst1Δhac1Δ* cells (p<0.0001), and that the corresponding double mutants had lifespan similar to that of wild-type cells (**Figure 2C**). In contrast, deletion of *IRE1* and *HAC1* did not significantly change the lifespan of the long-lived strain overexpressing Sir2 (SIR2OE) as well as *fob1Δ* and *tor1Δ* deletion mutants (**Figure S1B**, **C**, and **Table S2**). Therefore, these genetic epistasis experiments demonstrate that lifespan extension in the long-lived UPR target gene mutants is dependent on functional Ire1p and the ability to activate ER stress response. Moreover, deletion of UPR target genes extends lifespan by mechanisms distinct from those responsible for the lifespan extension observed under conditions of increased Sir2 activity or reduced mTOR signaling, a genetic mimic of dietary restriction [23].

### Elevated basal UPR activity in mutants lacking downstream components of the UPR does not confer resistance to pharmacologically induced ER stress

It is possible that constitutive activation of UPR signaling in the long-lived ER secretory pathway mutants may lead to increased resistance to pharmacologically induced ER stress. To test whether elevated basal UPR signaling may pre-condition cells against stress and increase cellular stress resistance, *alg12Δ* and *bst1Δ* strains were analyzed for growth in the presence of tunicamycin. However, both *alg12Δ* and *bst1Δ* had decreased resistance to this pharmacological ER stressor (**Figure 2D**) suggesting that *ALG12* and *BST1* deficiency puts cells at a disadvantage in the presence of ER stress. Moreover, the double mutant strains combining the long-lived deletions with either *ire1Δ* or *hac1Δ* completely abolished the growth of cells in the presence of tunicamycin, similar to *ire1Δ* and *hac1Δ* single mutants (**Figure S1D**). Together, these data indicate that Ire1p and Hac1p are required for lifespan extension in *alg12Δ* and *bst1Δ*, but the long lifespan in these mutants cannot be explained solely by increased ER stress resistance, at least as measured by tunicamycin resistance.

### Ribosome profiling detects translational control by UPR signaling

To characterize the mechanisms of lifespan extension in the long-lived ER secretory pathway mutants, we examined genome-wide translational changes in response to Ire1p hyperactivation in *alg12Δ* and *bst1Δ* mutants using ribosome profiling. Ribosome profiling is based on deep sequencing of ribosome-protected mRNA fragments and provides quantitative data on the translation level of thousands of genes [24]. A key advantage of this method is the much greater sensitivity than that obtained with microarrays as mRNA abundance is not always a good predictor of protein synthesis. When coupled with mRNA-sequencing (RNA-seq), ribosome profiling data can also be used to measure translational regulation by monitoring translation efficiency (TE).

We hypothesized that deletion of *ALG12* and *BST1* leads to activation of Ire1p and induction of cytoprotective pathways. To test if genes induced by *ALG12* and *BST1* deficiency are translationally regulated by the UPR, we first defined the list of UPR activated genes by measuring changes in mRNA abundance and protein translation in wild-type cells treated with tunicamycin. In *S. cerevisiae*, the UPR has been shown to transcriptionally activate ∼380 genes [10]. Many of these genes encode proteins that are components of secretory pathway organelles and are involved in translocation, protein folding, glycosylation, vesicular trafficking, and ERAD. We found that, following 30 min treatment with tunicamycin, translational changes were observed for 170 genes (changed more than 1.5-fold), of which 63 were down-regulated and 107 were up-regulated (**Table S3**). Genes up-regulated by tunicamycin treatment demonstrated a limited overlap with the genes whose expression was induced by the UPR as shown by microarray analysis [10] (**Figure S2A** and **Table S4**). As expected, many of the genes were regulated at the level of transcription, but our analysis also revealed a set of genes for which the scope of translational activation by the UPR was much greater compared to transcriptional induction (**Figure S2B**). Moreover, measuring translation rates allowed us to examine the relative contribution of translational regulation to both up-regulated and down-regulated changes. At the level of mRNA abundance, there were significantly fewer down-regulated genes (out of 241 genes that changed expression, 220 genes were induced and 21 were repressed) than genes repressed at the translational level (63 genes). The fact that there were more genes whose expression was reduced at the translational level indicates that UPR largely induces genes at the level of transcription, whereas UPR repressed genes are mostly regulated at the level of protein translation.

### Deletion of *ALG12* and *BST1* leads to transcriptional and translational induction of multiple stress response genes

**Figure 3:**
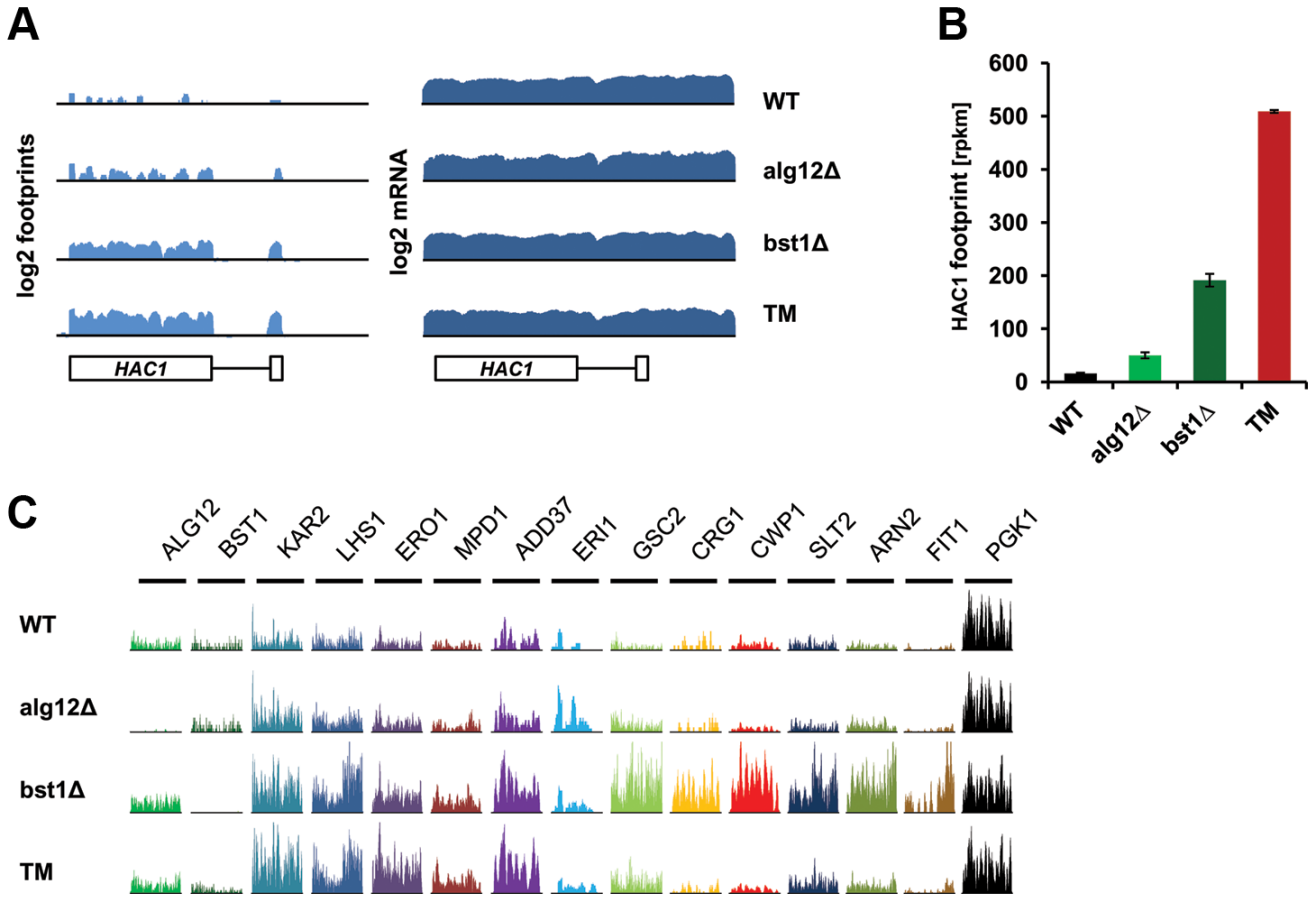
Deletion of *ALG12* and *BST1* induce UPR signaling and translation of UPR target genes. (**A**) Log_2_ footprints and mRNA (rpkm) for a region containing *HAC1* in untreated wild-type, *alg12Δ*, *bst1Δ*, and wild-type cells treated with tunicamycin (TM). (**B**) Footprint counts (rpkm) for *HAC1* in untreated wild-type, *alg12Δ*, *bst1Δ*, and wild-type cells treated with tunicamycin (TM). Error bars indicate SEM. Measurements from biological replicates are shown. (**C**) Ribosome footprint coverage for UPR target genes. The scales of the Y axis, which shows the number of footprint reads, are independent by gene.

We next used ribosome profiling to detect translational changes in the long-lived *alg12Δ* and *bst1Δ* mutants and found enhanced expression of UPR target genes, which correlated with increased *HAC1* mRNA splicing and production of Hac1p. We observed ∼3 and 12-fold increase in Hac1p production in *alg12Δ* and *bst1Δ*, respectively (**Figure 3A, B**). In the case of the *alg12Δ* mutant, more than 1.5-fold increase in protein production was observed for 34 genes, whereas 16 genes were down-regulated (**Table S5**). Compared to *alg12Δ*, *BST1* deficiency resulted in a much stronger translational regulation. In the *bst1Δ* mutant, translational changes were observed for 373 genes (52 genes were repressed and 321 genes were induced) (**Table S6**). As expected, there was a significant overlap with the genes that were up-regulated by tunicamycin treatment (**Figure S3**). Known UPR targets, including chaperones (*KAR2*, *LHS1*, *JEM1*, *SCJ1*), oxidoreductases (*ERO1*, *MPD1*, *EUG1*, *PDI1*) and genes involved in glycosylation (*PMT3*) and ERAD (*ADD37* and *HRD1*) were among the top hits (**Figure 3C** and **Table S5** and **S6**). In addition, genes involved in many other ER secretory pathway processes were induced including glycophospatidylinositol anchor synthesis (*ERI1*, *MCD4*, *GWT1*), lipid biogenesis (*INO4*, *SCS3*), and vesicular trafficking (*MVB12*, *ERV29*). Although many more of the UPR target genes were induced in the *bst1Δ* mutant compared to *alg12Δ*, the lower extent of induction in *alg12Δ* mutant cells can be explained by the lower level of ER stress and reduced *HAC1* splicing.

In addition to genes associated with secretory pathway function, both of the long-lived mutants showed enrichment in genes with functions in mRNA splicing and degradation (*CWC21*, *CWC25*, *DCS1*), iron homeostasis (*ARN2*, *FIT1*, *FIT3*, *FTH1*, *HMX1*, *TIS11/CTH2*), mitochondrial protein quality control and sorting (*MGR1*, *MSP1*), as well as multiple stress response pathways (*DDR2*, *HOR7*, *HLR1*, *LOT6*, *TSL1*, *DOG2*, *ICT1*, *SED1*, *CRG1*) [25]–[33].

**Figure 4:**
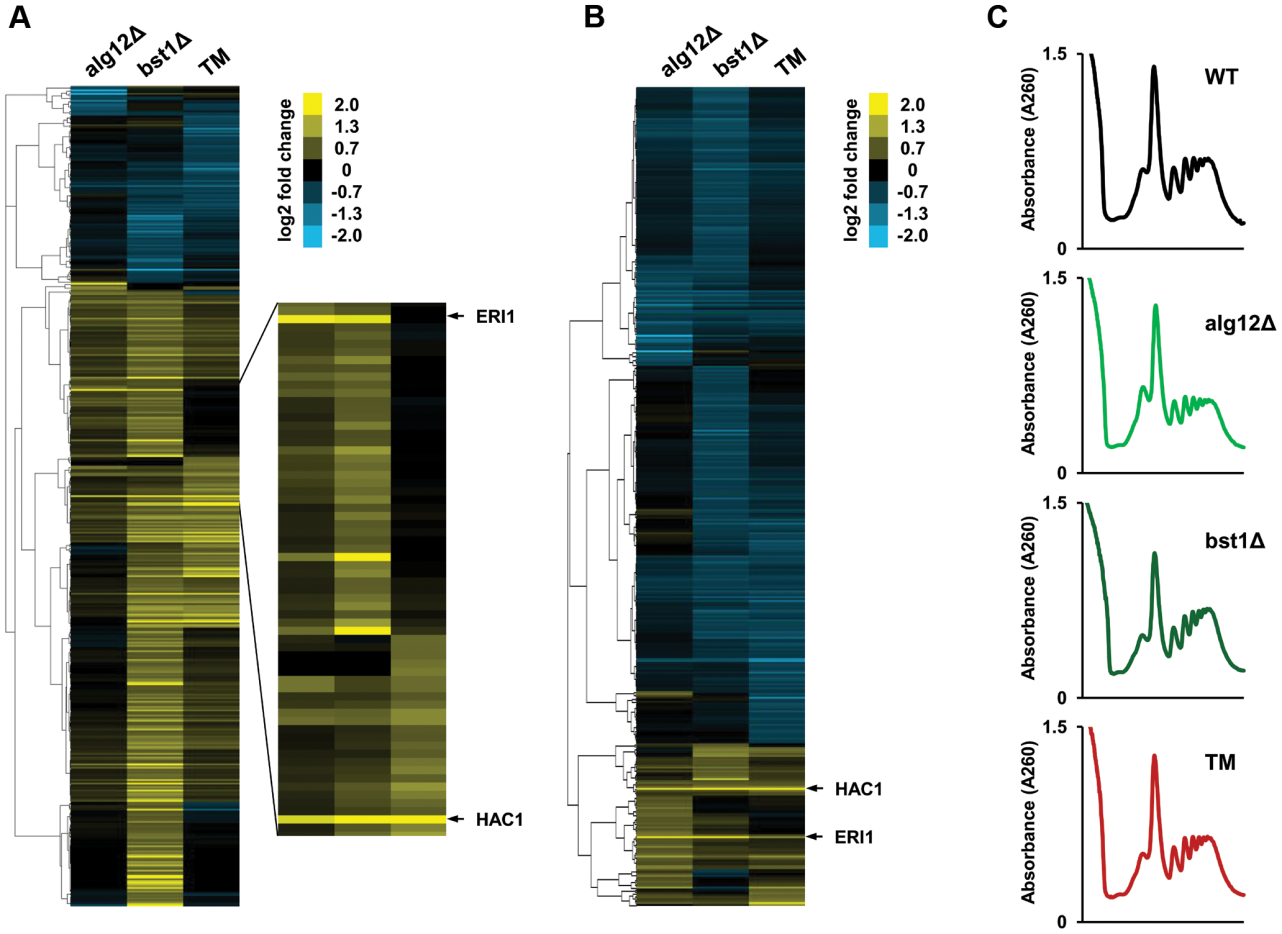
Translational control in the long-lived ER secretory pathway mutants. (**A**) Changes in protein translation in *alg12Δ*, *bst1Δ*, and wild-type cells treated with tunicamycin (TM) relative to untreated wild-type cells are shown in log_2_ scale for all genes that are activated or repressed more than 1.5-fold in at least one of the strains. (**B**) Cluster analysis of log2 TE changes in *alg12Δ*, *bst1Δ*, and TM treated cells relative to untreated wild-type cells. Changes in log2 TE are shown for all genes that showed more than 1.5-fold decrease or increase in TE. (**C**) Polysome profiles of *alg12Δ*, *bst1Δ*, and TM treated cells. Long-lived deletion strains *alg12Δ* and *bst1Δ* do not show overall translational suppression.

Similar to the tunicamycin treated cells, the number of genes whose translation was increased in *alg12Δ* and *bst1Δ* mutants exceeded the number of down-regulated genes (**Figure 4A**). To analyze if any of the observed differences can be explained by translational control, we calculated TE for each mRNA, which represents the relative number of footprints normalized to mRNA abundance. A significantly larger fraction of genes whose TE changed more than 1.5-fold had a decreased TE rather than increased (**Figure 4B**). These data are consistent with the overall down-regulation of protein synthesis during ER stress. However, several genes showed translational activation in *alg12Δ* and *bst1Δ* mutants as well as in tunicamycin treated cells. Among these genes, *HAC1* and *ERI1* were strongly regulated at the level of translation, but were not up-regulated transcriptionally.

### Induction of the cell wall integrity signaling in *alg12*Δ and *bst1*Δ

**Figure 5:**
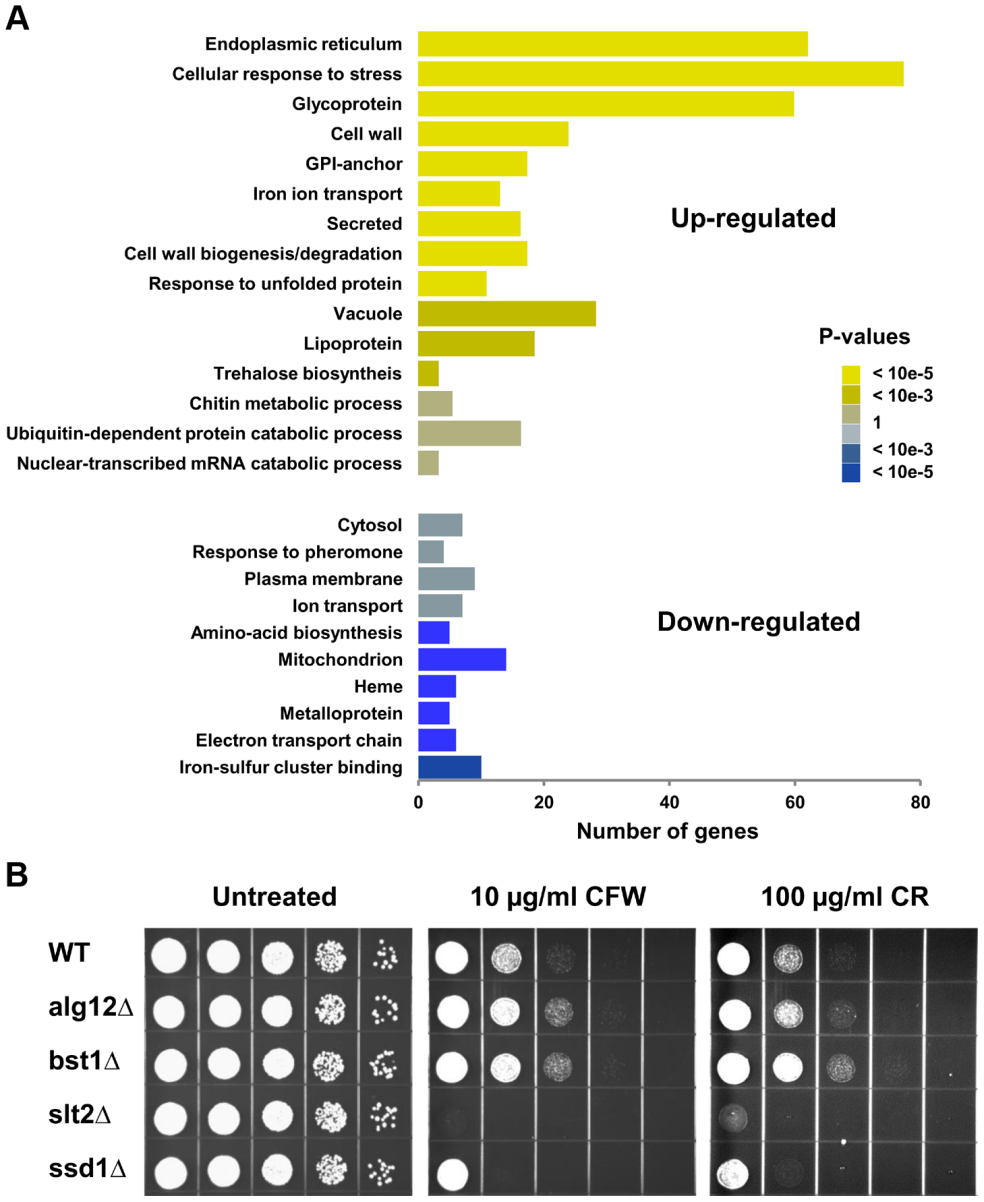
Deletion of *ALG12* and *BST1* leads to activation of the CWI signaling pathway. (**A**) Gene Ontology analysis of differentially regulated genes in *bst1Δ*. X-axis shows the number of genes in each functional category. (**B**) Sensitivity of *alg12Δ* and *bst1Δ* mutant strains to cell wall stress caused by calcofluor white (CFW) and congo red (CR). For each strain, 10× serial dilutions of logarithmically growing cells were spotted on agar plates containing indicated concentrations of the drugs. Pictures were taken after 48 h incubation at 30°C.

Gene ontology analyses (DAVID) [34] of footprint data revealed that a number of genes up-regulated in the long-lived mutants are involved in cellular response to stress (**Figure 5A**). A second cluster of genes that were expressed at higher level in *alg12Δ* and *bst1Δ* mutants comprises of cell wall components and genes involved in cell wall biogenesis. The induction of cell wall components in the long-lived strains was particularly appealing, as it suggested a link between the UPR, stress resistance and increased longevity. Many of the genes that code for proteins of the cell wall have been implicated in resistance to multiple stressors, and are known to be regulated by the cell wall integrity (CWI) pathway. The CWI pathway responds to cell wall stress through several cell-surface sensors (Wsc1p, Wsc2p, Wsc3p, Mid2p and Mtl1p) [35] that activate a small G protein, Rho1p. Activation of Rho1p triggers a MAPK signaling cascade leading to transcriptional up-regulation of CWI target genes through two transcription factors Rlm1p and SBF (Swi4p/Swi6p). Among other targets, CWI regulates synthesis of β-glucan and biogenesis of cell wall components. Strikingly, among genes that were up-regulated in the long-lived mutants were *MID2* stress sensor, *SLT2/MPK1* MAPK kinase, and *RLM1* transcription factor. We also observed increased expression of Rlm1p transcription factor targets in *bst1Δ* mutant including genes involved in cell wall biogenesis (β-glucan synthases *GSC2/FKS2* and *FKS1*, chitin synthase *CHS3*) and multiple cell wall components (*BGL2*, *CIS3*, *CWP1*, *CWP2*, *CRH1*, *SED1 YLR194C*) [36]. To test if the CWI pathway is important for the lifespan extension in *alg12Δ* and *bst1Δ* mutants, we tested sensitivity of these strains to calcofluor white and congo red, which are known pharmacological inducers of the cell wall stress. Consistent with activation of the CWI signaling, we observed increased resistance of both *alg12Δ* and *bst1Δ* to cell wall stress compared to wild-type strain (**Figure 5B**). Moreover, induction of the CWI signaling in the long-lived ER secretory pathway mutants was associated with increased resistance to other stresses including heat shock and oxidative stress (**Figure S4**), providing additional evidence that deletion of *ALG12* and *BST1* confers multiple stress resistance.

We also found strong induction of genes involved in trehalose (*TSL1*, *TPS1*, *TPS2*, *NTH1*) and chitin (*CHS1*, *CHS7*, *CRH1*, *GFA1*) synthesis. Increased trehalose and chitin accumulation is a common cell defense strategy that protects cells against a variety of stressful conditions, including heat, acid and cold shock. Another potential target up-regulated in *bst1Δ* that may mediate lifespan extension is glycerol-3-phosphate dehydrogenase *GPD1*. Gpd1 catalyzes the production and accumulation of glycerol in response to hyperosmotic stress and acts as an osmoregulator by preventing loss of water and turgor of the cells. Induction of the osmotic stress response and increased glycerol production have been shown to extend yeast replicative lifespan, whereas deletion of *GPD1* shortens lifespan even in the absence of osmotic stress [37]. In addition, up-regulation of glycerol biosynthesis genes has been linked to extension of chronological lifespan in Tor1- and Sch9-deficient mutants [38].

Decreased protein translation has been shown to extend lifespan in a wide range of species, including *S. cerevisiae*, *Caenorhabditis elegans*, and *Drosophila melanogaster* [39], [40]. For example, increased longevity caused by Tor1p inhibition or knockout of Tor1-regulated *SCH9* kinase is achieved, at least in part, by reduction in mRNA translation. In addition, decreased protein synthesis caused by deficiency of ribosomal protein subunits often leads to ER stress resistance and increased lifespan [15]. However, overall protein translation was not affected in *alg12Δ* and *bst1Δ* mutants (**Figure 4C**). We also did not observe changes in the expression of antioxidant genes or components of proteasome suggesting that elevated proteasomal capacity and oxidative stress response do not contribute to longevity in *alg12Δ* and *bst1Δ* mutants. In addition, we did not observe induction of other stress response transcription factors, including *YAP1*, *SKN7*, *MSN2* and *MSN4*.

Taken together, our data demonstrate that lifespan extension conferred by the ER secretory pathway mutants is dependent on functional UPR, and that increased basal UPR signaling may promote longevity in *S. cerevisiae* through increased expression of multiple stress response genes and activation of the CWI-MAPK pathway.

## Discussion

It is commonly accepted that aging is associated with a decline in homeostatic mechanisms that protect organisms from accumulation of senescence factors including aggregated proteins, oxidatively damaged cellular components and toxic metabolites [41]–[43]. Recent studies suggest that cellular capacity to adapt to ER stress may also decline with age [44]. Cells respond to accumulation of misfolded proteins in the ER by activating the UPR signaling pathway that restores ER homeostasis by degrading misfolded proteins, inhibiting translation, and increasing expression of chaperones and oxidative folding components [1]. Although the mechanisms by which cells sense ER stress and activate stress response genes are well studied [45], [46], the role of UPR signaling in aging remains unknown. We have begun to characterize the role of UPR in regulating lifespan in *S. cerevisiae*. To our surprise, we determined that inactivation of *IRE1* and *HAC1* that are involved in sensing ER stress in yeast does not affect lifespan under physiological conditions. However, from the analysis of 14 different UPR target gene deletions, at least 9 were found to be significantly long-lived. In addition, we found that extended lifespan in the UPR target gene deletion mutants is associated with increased basal UPR activity. These observations prompted us to hypothesize that deletion of genes downstream of UPR may lead to constitutive activation of Ire1p and increased ER stress resistance. Our data provide evidence that functional Ire1p and transcriptional factor Hac1p are required for lifespan extension by deletion of UPR target genes.

Despite elevated basal UPR activity in *alg12Δ* and *bst1Δ*, these strains were not resistant to pharmacologically induced ER stress conferred by tunicamycin. This provides an interesting contrast to another recent study whereby it was found that many ribosomal deletion mutants were resistant to tunicamycin through a *HAC1*-independent mechanism [15]. In that case, however, tunicamycin resistance did not correlate with lifespan extension. From comparing these studies, it is clear that the long lifespan of *alg12Δ* and *bst1Δ* does not come from mitigating ER stress, at least phenocopied by tunicamycin exposure, and that induction of the UPR and associated stress response pathways is more likely to modulate aging through separate mechanisms.

We used ribosome profiling to identify specific pathways and protective mechanisms that contribute to lifespan extension in the long-lived ER secretory pathway mutants at the genome-wide level. Using this method, we identified translational changes in the long-lived mutants *alg12Δ* and *bst1Δ* compared to wild-type cells in unstressed conditions. We discovered that *ALG12* and *BST1* deficiency selectively regulates a subset of genes that belong to only a few functional groups. In addition to activation of UPR target genes, we observed induction of other cytoprotective pathways including general stress response proteins and proteins involved in multidrug resistance. The second most prominent change that occurs in the long-lived ER secretory pathway mutants is cell wall remodeling. Although multiple signaling pathways contribute to remodeling of the cell wall, the regulation of this process is controlled primarily by the mitogen-activated protein kinase (MAPK) Slt2p/Mpk1p via the CWI pathway. Our analysis revealed extensive up-regulation of components of CWI signaling including Slt2p/Mpk1p, Mid2p cell wall stress sensor and Rlm1p transcription factor. Activation of the CWI pathway leads to an increased synthesis of β-glucan and enhanced expression of Rlm1p targets that confer resistance to multiple stresses. Genes up-regulated by CWI signaling have been implicated in the tolerance of *S. cerevisiae* to a variety of stressors including oxidative stress, heat shock, hypo-osmotic stress, actin depolymerization, high and low pH stress and DNA damage [35]. In addition to the CWI signaling cascade, two Slt2p-independent pathways, which require Mpt5p and Ssd1p, have been shown to regulate integrity of the cell wall and promote longevity in *S. cerevisiae* [47]. Interestingly, *MPT5* and *SSD1* encode RNA-binding proteins that have been proposed to post-transcriptionally up-regulate expression of genes involved in cell wall biogenesis by increasing TE and stability of the target mRNAs. We found significant up-regulation of Ssd1 in at least one of the long-lived ER secretory pathway mutants analyzed in our study (*bst1Δ*). Therefore, we conclude that the lifespan extension in the ER secretory pathway mutants does not result solely from improved protein homeostasis caused by UPR activation, but might also require activation of multiple stress response pathways.

In support of this mechanism, we identified four components of the chitin biosynthesis (*CHS1*, *CHS7*, *CRH1*, *GFA1*) and four genes involved in the synthesis of trehalose (*TSL1*, *TPS1*, *TPS2*, *NTH1*). Chitin, β(1,4)-linked N-acetylglucosamin polymer, serves as a structural component of the cell wall and represents about 1–2% of its inner layer polymers. However, during stress, cell wall chitin levels can increase up to 20% [48] making cell tolerant to adverse environmental conditions. In turn, trehalose (α,α-glucose disaccharide) has been implicated in heat shock resistance. In response to thermal stress, accumulation of cytoplasmic trehalose leads to increased osmolarity that protects proteins from denaturation and aggregation [49].

Another possible means by which deletion of UPR transcriptional targets could increase replicative lifespan is by enhancing mitochondrial protein turnover. Two other genes that were up-regulated in *bst1Δ* mutant include *MGR1* and *MSP1*. *MGR1* encodes a component of the mitochondrial inner-membrane iAAA protease complex that functions to degrade misfolded mitochondrial proteins and participates in protein quality control in mitochondria [50], whereas Msp1p is involved in mitochondrial protein sorting [51]. Thus, activation of mitochondrial protein turnover upon ER stress caused by deletion of UPR components may increase longevity in part by influencing mitochondrial function.

Activation of the UPR signaling in the UPR target gene deletion mutants is consistent with previous reports showing hyperactivation of UPR in cells lacking genes involved in the ER secretory pathway function. In yeast, deletion of several components of the ERAD system (*DER1*, *SEL1*, *HRD1*, *UBC1* and *UBC7*) [10], [52] as well as inactivation of the ER chaperone Kar2p [53] have been shown to dramatically induce UPR. More recently, hundreds of single-gene knockouts have been shown to perturb UPR signaling in yeast representing a number of diverse functional groups [20]. It would be important to determine in future studies if these deletion mutants show significant overlap with those affecting longevity and the requirement for increased UPR function in these settings.

Although stress resistance correlates with increased longevity in a variety of model organisms, including yeast [15], [18], worms [54], [55] and fruit flies, the link of UPR signaling pathway, ER stress resistance and longevity remains poorly understood. Interestingly, inactivation of IRE-1 and XBP-1 results in shortened lifespan in *C. elegans*, and UPR signaling contributes to the increased longevity of *daf-2* mutants and in response to dietary restriction [56], [57]. Moreover, overexpression of a constitutively active form of XBP-1 in neurons, but not in other tissues, results in increased ER stress resistance and extends lifespan in worms [58]. However, ubiquitous up-regulation of UPR signaling in the whole animal does not promote longevity despite elevated resistance to ER stressors.

Our data suggest that while increased UPR signaling is an important determinant of lifespan extension, it is not sufficient to confer enhanced ER stress resistance in yeast cells. Instead, we found that the increased longevity in the UPR target gene mutants is associated with induction of multiple stress response programs. Taken together, these data highlight the complexity of organism's response to various stresses and demonstrate interdependencies among multiple longevity pathways.

## Materials and Methods

### Yeast strains and media

All yeast strains were derived from the parent strains of the haploid yeast ORF knockout collection [59], BY4741 (*MAT* ***a*** *his3Δ1 leu2Δ0 met15Δ0 ura3Δ0*) and BY4742 (*MATα his3Δ1 leu2Δ0 lys2Δ0 ura3Δ0*), or the DAmP library [60] (**Table S7**). Double mutant strains combining the long-lived deletions with either *ire1Δ* or *hac1Δ* were prepared by standard PCR-based gene disruption method. The deletion of each ORF was confirmed by PCR with locus-specific primers (**Figure S5**). All strains were grown at 30°C in complete YPD medium (1.0% yeast extract, 2.0% peptone, and 2.0% glucose).

### Replicative lifespan assay

Lifespan assays were carried out as described previously [61]. Analysis of replicative lifespan is based on the ability of budding yeast to divide asymmetrically producing distinct mother and daughter cells. For the replicative lifespan assay, cells were grown on freshly prepared YPD plates for 2 days at 30°C. For each strain, founder cells were plated on agar plates by selecting the newborn daughter cells using micromanipulator. Cells were monitored for cell divisions every 90 min, and subsequent budded daughter cells were separated and removed as they formed. The process continued until cells stopped dividing. Replicative lifespan was calculated as the number of times each mother cell divided before it underwent senescence. Statistical analysis of the lifespan data was performed using a Wilcoxon Rank-Sum test.

### *HAC1* mRNA splicing

Total RNA was extracted by RiboPure-Yeast Kit (Ambion) according to the manufacturer's instructions. RNA was treated with DNaseI, and first strand cDNA was synthesized using the SuperScript III reverse transcriptase (Invitrogen) with random hexamer primers. For the analysis of *HAC1* mRNA slicing, RT-PCR was performed with the following primers that flank the *HAC1* intron: 5′-CCGTAGACAACAACAATTTG-3′ and 5′-CATGAAGTGATGAAGAAATC-3′. PCR fragments were resolved on 2% agarose gels, stained with EtBr, and quantified by densitometry.

### Spot assays

Resistance of strains to tunicamycin, calcofluor white, and congo red was determined using spot assays. Cells were initially grown in liquid culture without the drugs until OD_600_ = 0.6, and 10× serial dilutions for each strain were spotted on agar plates containing indicated concentrations of the drugs. The plates were incubated at 30°C, and images were taken 48 h after plating.

### Ribosome profiling and mRNA sequencing

Yeast cultures were grown to OD_600_ = 0.5 in 500 ml of complete YPD medium, and cells were collected by filtering through 0.45 µm filter (Millipore) with glass holder. Pellets were scraped with spatula, flash frozen in liquid nitrogen and stored at −80°C. To pharmacologically induce ER stress, tunicamycin was added into the medium at a final concentration 1 µg/ml, and cells were incubated at 30°C for an additional 30 min. Yeast extracts were prepared by cryogrinding the cell paste with BioSpec cryomill. Aliquots of cell lysates were used for footprint extraction and isolation of total RNA. Preparation of lysates, ribosome fractionation, and construction of footprint and RNA-seq libraries were performed as in [62] with modifications. A detailed description of protocols can be found in the **Text S1**. Sequencing of footprint and RNA-seq libraries was performed on the Illumina HiSeq2000 platform. Primers used in library preparation are listed in **Table S8**.

### Bioinformatics analyses

Ribosomal footprints and mRNA reads were aligned to the *S. cerevisiae* genome from the Saccharomyces Genome Database (SGD, http://www.yeastgenome.org/, release number R64-1-1). Sequence alignment was performed by Bowtie software v.0.12.7 [63] allowing two mismatches per read. Custom Perl scripts were used to count reads over features of interests (genes, UTRs etc.), deal with introns, overlaps and highly homologous sequences.

### Analyses of differential gene translation and translation efficiency

To analyze differential gene expression and translation we disregarded 100 nt from the 5′-end of each gene therefore avoiding bias caused by the region with elevated footprint density in the vicinity of the ATG start codon. Rpkm (reads per kilobase per million mapped reads) values, which represent the number of reads normalized to gene length and total number of reads, were used as a measure of gene expression. An average rpkm value for two biological replicates was calculated for each gene, and the genes with fewer than 10 rpkm were excluded from further analysis (**Figure S6**). The gene was considered regulated if its rpkm value changed more than 1.5-fold (0.6 in log2 scale). To calculate TE, footprint rpkm values were divided by mRNA rpkm. Clustering was performed using Cluster 3.0 software [64] and the data were visualized using Java Treeview [65].

### Polysome analyses

Polysome profile analysis of an aliquot of cell extracts was performed using sucrose gradients (10–50% wt/wt) in polysome gradient buffer [20 mM TrisHCl (pH 8.0), 140 mM KCl, 5 mM MgCl_2_, 0.2 g/l cycloheximide, 0.5 mM DTT]. 1 ml of cell lysate containing 50 units (OD_260_) were loaded on top of the gradients, and sedimented at 35,000 rpm at 4°C in a SW41 Ti rotor (Beckman) for 3 h. Gradients were collected from the top using the Brandel gradient fractionation system and profiles were monitored at 254 nm.

## Supporting Information

Figure S1 The ER secretory pathway mutants extend lifespan by mechanisms distinct from those in SIR2OE and *fob1Δ*. (**A**) Analysis of Kar2p expression in the ER secretory pathway mutants. (**B, C**) *SIR2* overexpression (SIR2OE) and deletion of *FOB1* (*fob1Δ*) extend yeast replicative lifespan by mechanisms independent of Ire1p and Hac1p. Mean lifespans are shown in parentheses. (**D**) Sensitivity of the *ire1Δ* and *hac1Δ* mutants to ER stress. For each strain 10× serial dilutions of logarithmically growing cells were spotted on agar plates without the drug (untreated) or plates containing 0.2 µg/ml tunicamycin (TM). Pictures were taken after 48 h incubation at 30°C.
(TIF)

Figure S2 Analysis of coordinate changes in translation and transcription in response to ER stress. (**A**) Comparison of genes up-regulated by tunicamycin from this study with known UPR targets. Treatment of wild-type cells with tunicamycin (TM) induced protein translation more than 1.5-fold for 107 genes. Known transcriptional UPR targets include 381 genes as defined by Travers et al. [10]. These two groups have 43 genes in common. (**B**) Comparison of genes whose mRNA abundance and protein translation were induced by tunicamycin treatment.
(TIF)

Figure S3 Regulation of protein translation in the long-lived ER secretory pathway mutants. (**A**) Genes whose protein translation was induced more than 1.5-fold in *alg12Δ* and *bst1Δ* mutants and tunicamycin (TM) treated wild-type cells. (**B**) Genes whose protein translation was repressed more than 1.5-fold in *alg12Δ* and *bst1Δ* mutants and tunicamycin (TM) treated wild-type cells.
(TIF)

Figure S4 Deletion of *ALG12* and *BST1* leads to heat and oxidative stress resistance in yeast. (**A**) Sensitivity of *alg12Δ* and *bst1Δ* mutant strains to heat stress. Ten-fold serial dilutions of log-phase cultures were plated onto YPD and incubated at either 30° or 40°C, and images were taken 48 h after plating. (**B**) Viability of *alg12Δ* and *bst1Δ* mutant strains following treatment with 1 mM hydrogen peroxide (H_2_O_2_) for 30 min. Results are represented as means ± SEM from three independent experiments.
(TIF)

Figure S5 Verification of mutant strains prepared in this study. (**A, B**) Double mutant strains combining *alg12Δ* and *bst1Δ* deletions with either *ire1Δ* or *hac1Δ* were verified by PCR analysis with locus-specific primers.
(TIF)

Figure S6 Reproducibility of footprint and mRNA-abundance measurements. (**A, B**) Comparison of footprint and mRNA-abundance measurements between two replicates. Footprint and mRNA rpkm values are shown for wild-type cells treated with tunicamycin. Pearson correlation coefficients are indicated.
(TIF)

Table S1 Replicative lifespan analysis of ER stress response gene deletion strains.
(XLS)

Table S2 Deletion of *IRE1* and *HAC1* prevents lifespan extension in *alg12Δ* and *bst1Δ* mutants, but not in SIR2OE, *fob1Δ* and *tor1Δ* mutants.
(DOCX)

Table S3 Genes whose footprint coverage was changed more than 1.5-fold by tunicamycin treatment.
(XLSX)

Table S4 Comparison of genes up-regulated by tunicamycin from this study with targets of the UPR as defined by Travers et al. [10].
(XLSX)

Table S5 Genes whose footprint coverage was changed more than 1.5-fold by *ALG12* deficiency.
(XLSX)

Table S6 Genes whose footprint coverage was changed more than 1.5-fold by *BST1* deficiency.
(XLSX)

Table S7 Strains used in this study.
(DOCX)

Table S8 Primers used for footprint and mRNA library preparation.
(DOCX)

Text S1 Supplementary text extending Materials and Methods.
(DOCX)
