# Supplementary figures and images for: Lifespan Extension Conferred by Endoplasmic Reticulum Secretory Pathway Deficiency Requires Induction of the Unfolded Protein Response
Source: PLoS Genet. 2014 Jan 2;10(1):e1004019. doi: 10.1371/journal.pgen.1004019 (PMC3879150; doi:10.1371/journal.pgen.1004019)

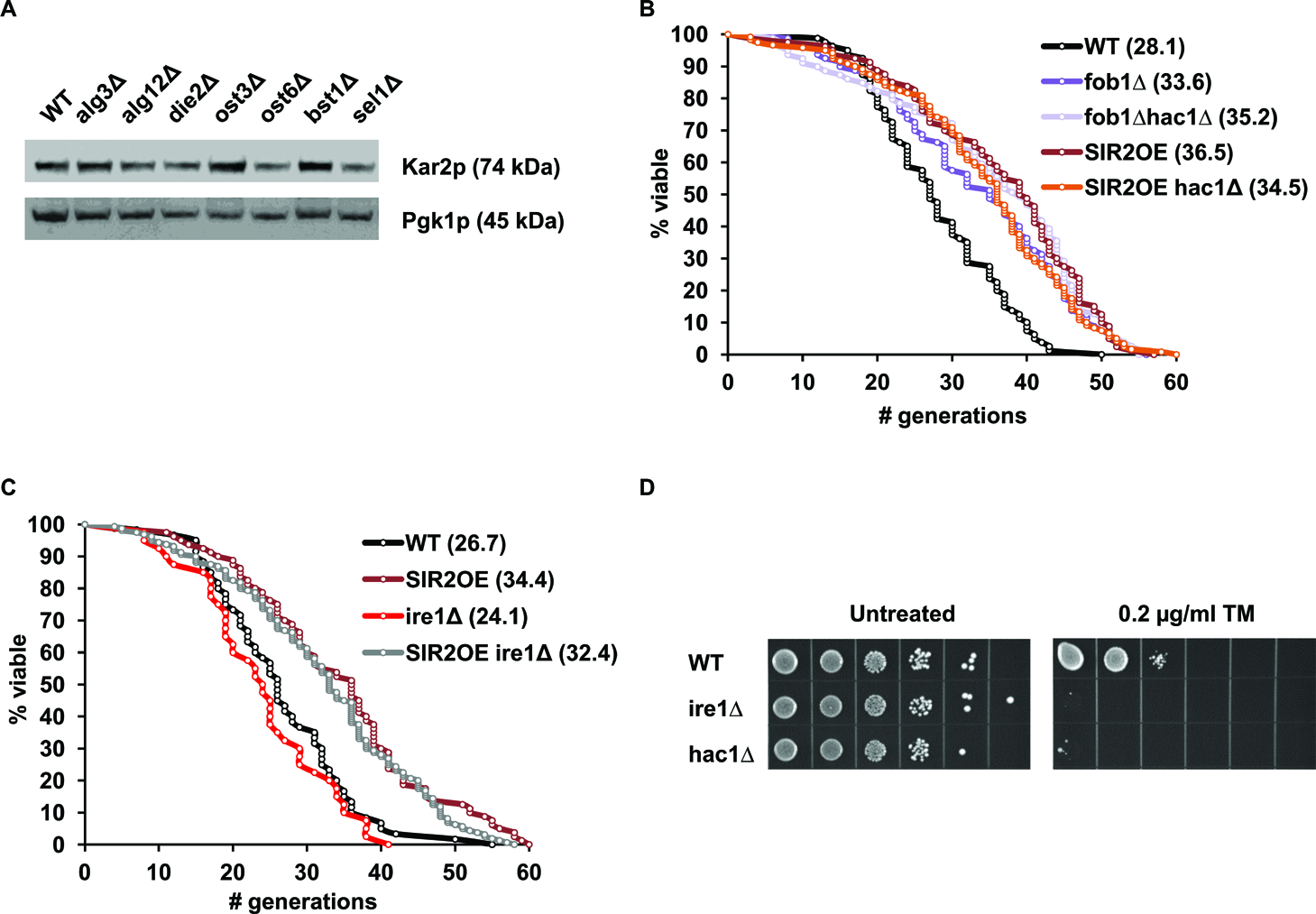

Supplement: Figure S1 — The ER secretory pathway mutants extend lifespan by mechanisms distinct from those in SIR2OE and fob1Δ. (A) Analysis of Kar2p expression in the ER secretory pathway mutants. (B, C) SIR2 overexpression (SIR2OE) and deletion of FOB1 (fob1Δ) extend yeast replicative lifespan by mechanisms independent of Ire1p and Hac1p. Mean lifespans are shown in parentheses. (D) Sensitivity of the ire1Δ and hac1Δ mutants to ER stress. For each strain 10× serial dilutions of logarithmically growing cells were spotted on agar plates without the drug (untreated) or plates containing 0.2 µg/ml tunicamycin (TM). Pictures were taken after 48 h incubation at 30°C. (TIF) [file pgen.1004019.s001.tif]

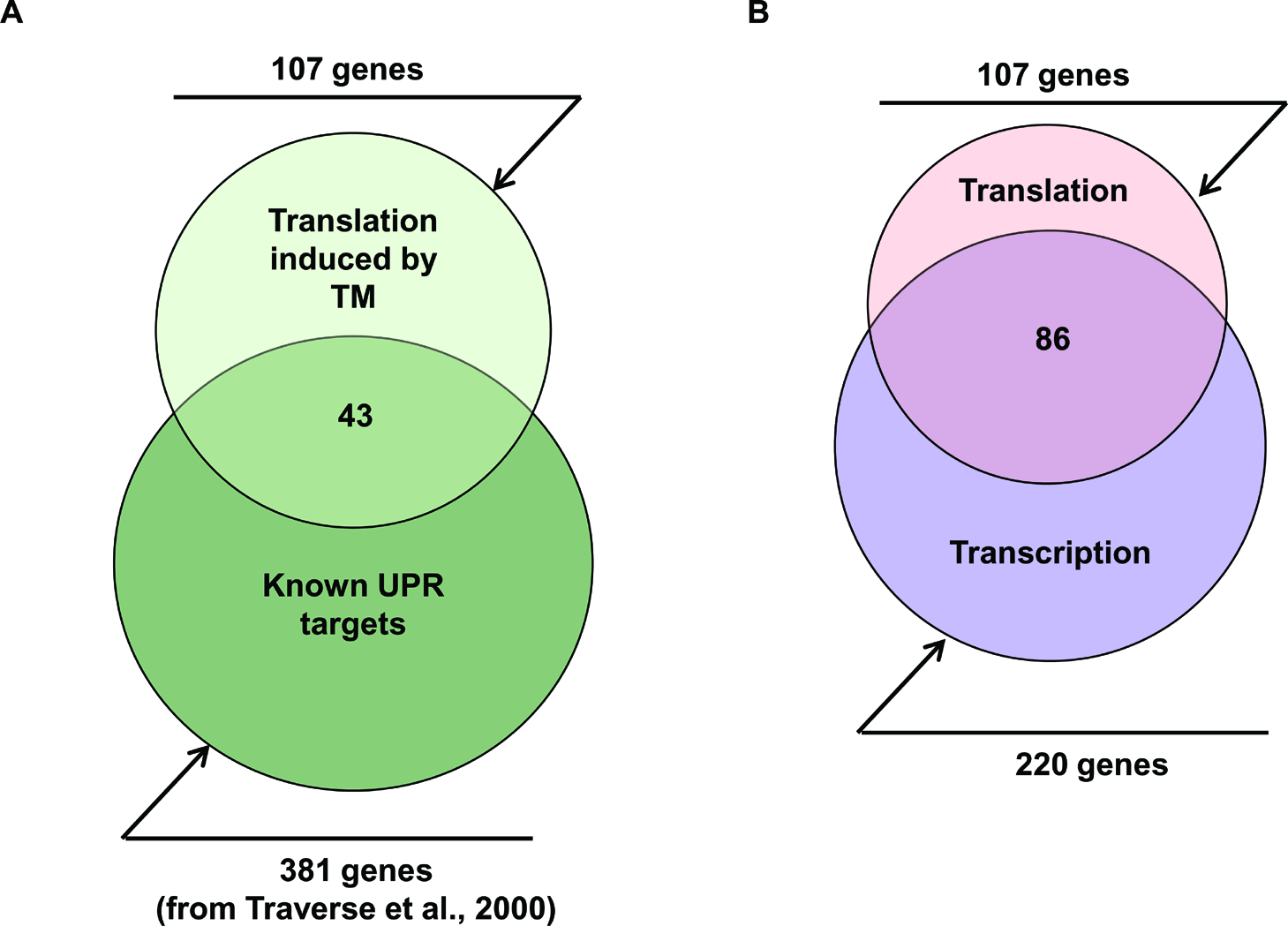

Supplement: Figure S2 — Analysis of coordinate changes in translation and transcription in response to ER stress. (A) Comparison of genes up-regulated by tunicamycin from this study with known UPR targets. Treatment of wild-type cells with tunicamycin (TM) induced protein translation more than 1.5-fold for 107 genes. Known transcriptional UPR targets include 381 genes as defined by Travers et al. [10]. These two groups have 43 genes in common. (B) Comparison of genes whose mRNA abundance and protein translation were induced by tunicamycin treatment. (TIF) [file pgen.1004019.s002.tif]

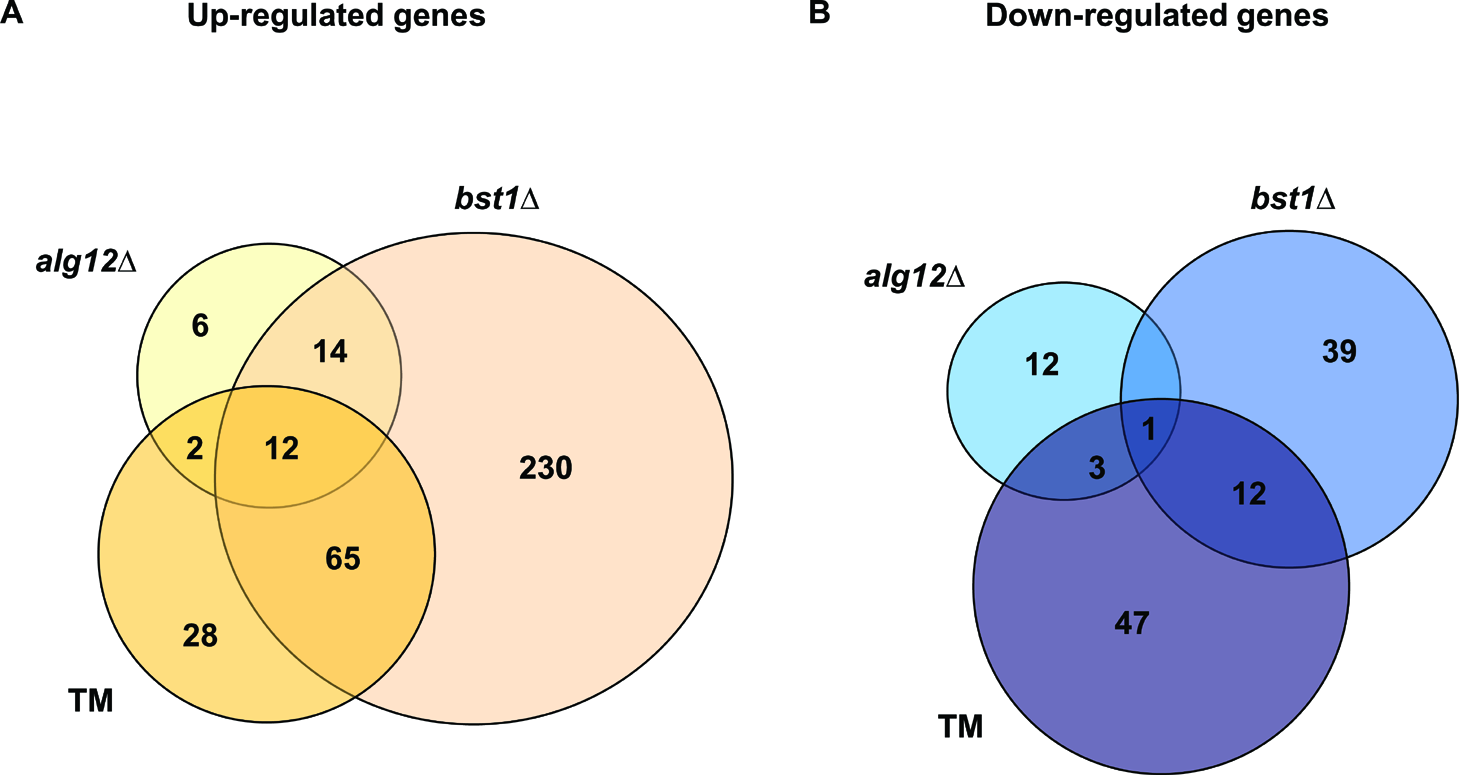

Supplement: Figure S3 — Regulation of protein translation in the long-lived ER secretory pathway mutants. (A) Genes whose protein translation was induced more than 1.5-fold in alg12Δ and bst1Δ mutants and tunicamycin (TM) treated wild-type cells. (B) Genes whose protein translation was repressed more than 1.5-fold in alg12Δ and bst1Δ mutants and tunicamycin (TM) treated wild-type cells. (TIF) [file pgen.1004019.s003.tif]

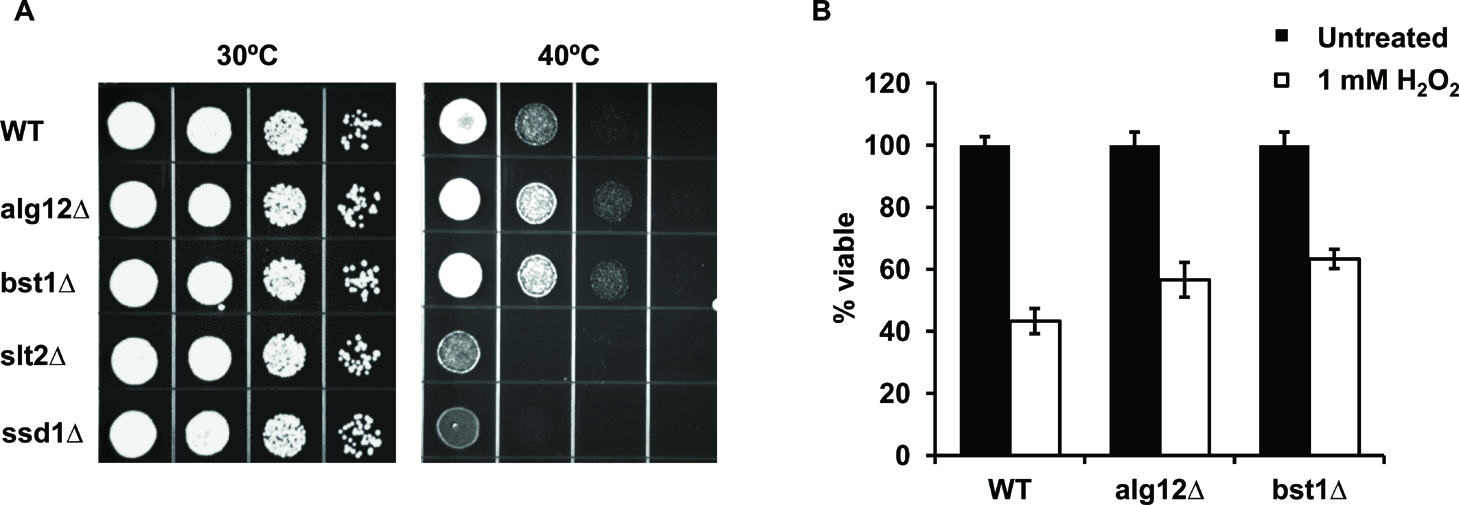

Supplement: Figure S4 — Deletion of ALG12 and BST1 leads to heat and oxidative stress resistance in yeast. (A) Sensitivity of alg12Δ and bst1Δ mutant strains to heat stress. Ten-fold serial dilutions of log-phase cultures were plated onto YPD and incubated at either 30° or 40°C, and images were taken 48 h after plating. (B) Viability of alg12Δ and bst1Δ mutant strains following treatment with 1 mM hydrogen peroxide (H2O2) for 30 min. Results are represented as means ± SEM from three independent experiments. (TIF) [file pgen.1004019.s004.tif]

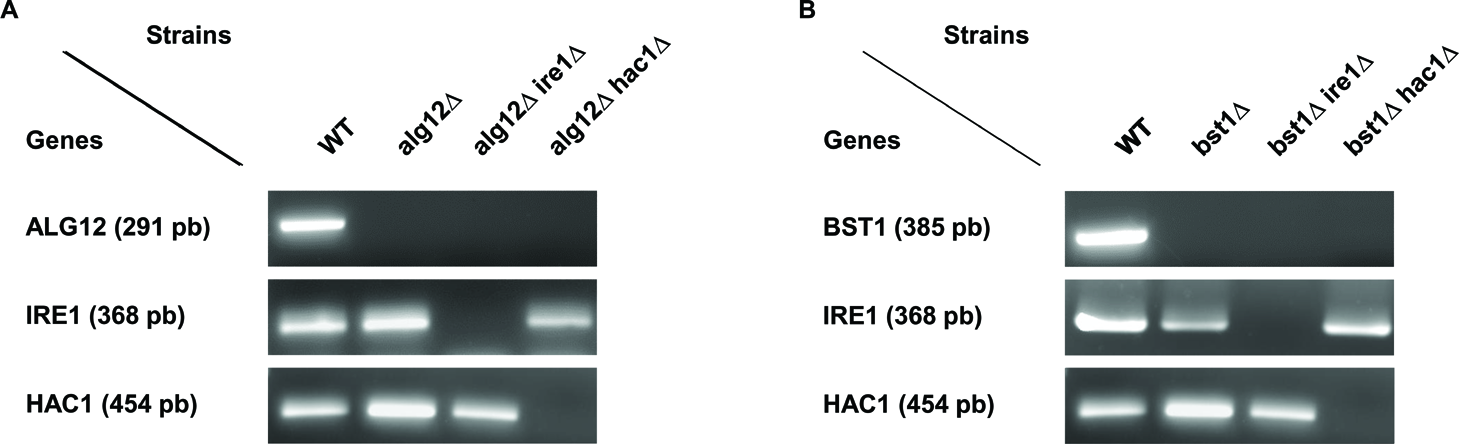

Supplement: Figure S5 — Verification of mutant strains prepared in this study. (A, B) Double mutant strains combining alg12Δ and bst1Δ deletions with either ire1Δ or hac1Δ were verified by PCR analysis with locus-specific primers. (TIF) [file pgen.1004019.s005.tif]

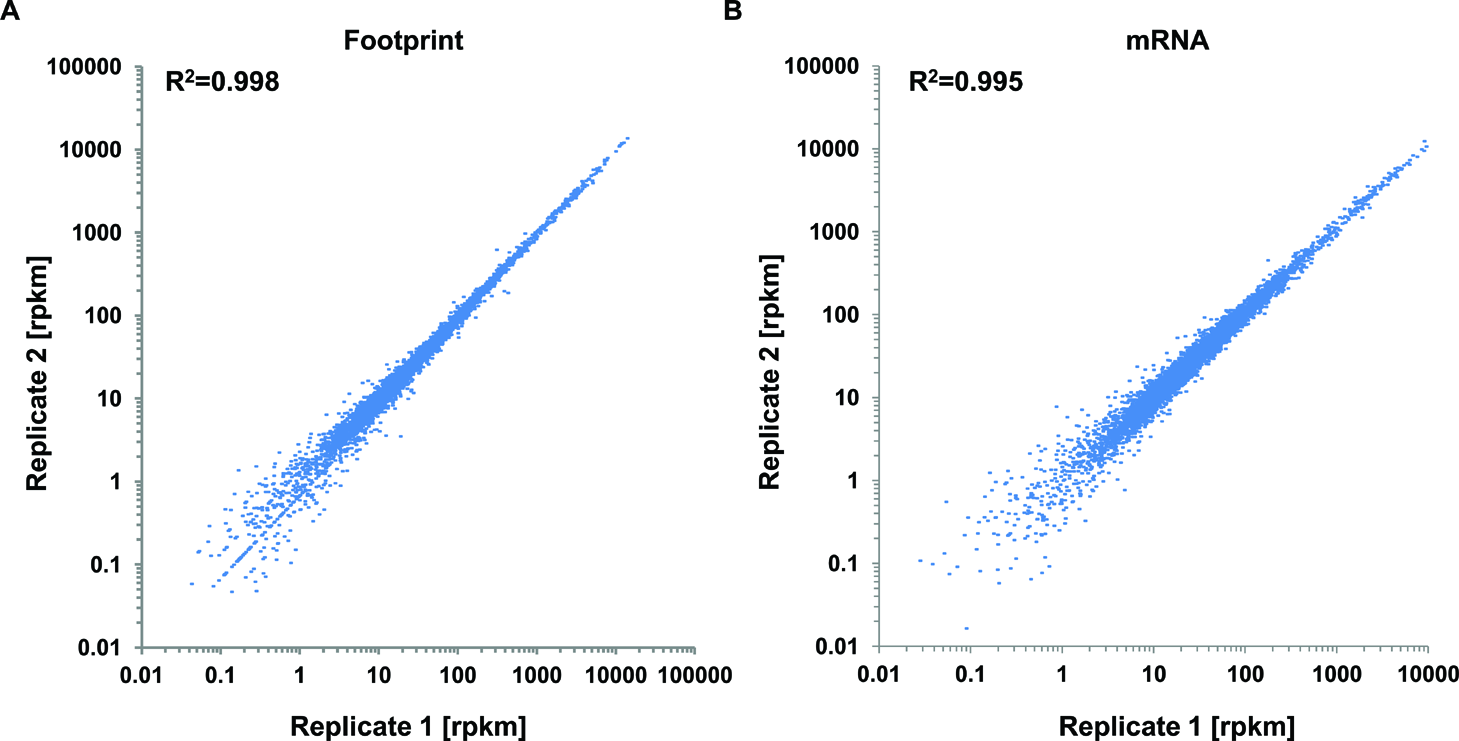

Supplement: Figure S6 — Reproducibility of footprint and mRNA-abundance measurements. (A, B) Comparison of footprint and mRNA-abundance measurements between two replicates. Footprint and mRNA rpkm values are shown for wild-type cells treated with tunicamycin. Pearson correlation coefficients are indicated. (TIF) [file pgen.1004019.s006.tif]
