# Supplementary material for: Lifespan Extension Conferred by Endoplasmic Reticulum Secretory Pathway Deficiency Requires Induction of the Unfolded Protein Response
Source: PLoS Genet. 2014 Jan 2;10(1):e1004019. doi: 10.1371/journal.pgen.1004019 (PMC3879150; doi:10.1371/journal.pgen.1004019)
Supplement: Table S2 — Deletion of IRE1 and HAC1 prevents lifespan extension in alg12Δ and bst1Δ mutants, but not in SIR2OE, fob1Δ and tor1Δ mutants. (DOCX) [file pgen.1004019.s008.docx]

**Table S2. Deletion of *IRE1* and *HAC1* prevents lifespan extension in *alg12∆* and *bst1∆* mutants, but not in SIR2OE, *fob1∆* and *tor1∆* mutants**

| **Deletion** | | |  | **Experiment-matched Control** | | | **% Mean RLS Change** | **Ranksum P-Value** |
| --- | --- | --- | --- | --- | --- | --- | --- | --- |
| **Genotype** | **Mean**  **RLS** | **N** |  | **Genotype** | **Mean**  **RLS** | **N** |  |  |
| *alg12Δ* | 28.2 | 100 |  | BY4741 | 24.0 | 100 | 17.4 | 0.0010 |
| *alg12Δire1Δ* | 17.1 | 100 |  | BY4741 | 24.0 | 100 | -29.0 | < 0.0001 |
| *alg12Δhac1Δ* | 18.5 | 100 |  | BY4741 | 24.0 | 100 | -23.2 | < 0.0001 |
| *bst1Δ* | 30.6 | 80 |  | BY4741 | 25.7 | 80 | 19.1 | < 0.0001 |
| *bst1Δire1Δ* | 23.9 | 80 |  | BY4741 | 25.7 | 80 | -7.0 | 0.1664 |
| *bst1Δhac1Δ* | 24.1 | 80 |  | BY4741 | 25.7 | 80 | -6.2 | 0.1470 |
| *fob1Δ* | 33.6 | 80 |  | BY4742 | 28.1 | 80 | 19.6 | 0.0009 |
| *fob1Δhac1Δ* | 35.2 | 79 |  | BY4742 | 28.1 | 80 | 25.3 | < 0.0001 |
| SIR2OE | 36.5 | 80 |  | BY4742 | 28.1 | 80 | 29.9 | < 0.0001 |
| SIR2OE *hac1Δ* | 34.5 | 120 |  | BY4742 | 28.1 | 80 | 22.8 | < 0.0001 |
| SIR2OE | 34.4 | 80 |  | BY4742 | 26.7 | 60 | 28.8 | < 0.0001 |
| *ire1Δ* | 24.1 | 40 |  | BY4742 | 26.7 | 60 | -9.7 | 0.2496 |
| SIR2OE *ire1Δ* | 32.4 | 160 |  | BY4742 | 26.7 | 60 | 21.3 | 0.0004 |
| *tor1Δhac1Δ* | 33.1 | 220 |  | BY4742 | 29.6 | 180 | 22.9 | < 0.0001 |
