# Supplementary material for: Lifespan Extension Conferred by Endoplasmic Reticulum Secretory Pathway Deficiency Requires Induction of the Unfolded Protein Response
Source: PLoS Genet. 2014 Jan 2;10(1):e1004019. doi: 10.1371/journal.pgen.1004019 (PMC3879150; doi:10.1371/journal.pgen.1004019)
Supplement: Table S7 — Strains used in this study. (DOCX) [file pgen.1004019.s013.docx]

**Table S7. Strains used in this study.**

| **Strain** | **Designation** | **Genotype** |
| --- | --- | --- |
| BY4741 | WT | MATa *his3 leu2 met15 ura3* |
| VL001 | *ire1Δ* | MATa *his3 leu2 met15 ura3 ire1Δ::KAN* |
| VL002 | *hac1Δ* | MATa *his3 leu2 met15 ura3 hac1Δ::KAN* |
| VL003 | *hrd1Δ* | MATa *his3 leu2 met15 ura3 hrd1Δ::KAN* |
| VL004 | *pdi1*-DAmP | MATa *his3 leu2 met15 ura3 pdi1-DAmP::KAN* |
| VL005 | *kar2*-DAmP | MATa *his3 leu2 met15 ura3 kar2-DAmP::KAN* |
| VL006 | *ost6Δ* | MATa *his3 leu2 met15 ura3 ost6Δ::KAN* |
| VL007 | *sel1Δ* | MATa *his3 leu2 met15 ura3 sel1Δ::KAN* |
| VL008 | *ost3Δ* | MATa *his3 leu2 met15 ura3 ost3Δ::KAN* |
| VL009 | *mpd1Δ* | MATa *his3 leu2 met15 ura3 mpd1Δ::KAN* |
| VL010 | *alg3Δ* | MATa *his3 leu2 met15 ura3 alg3Δ::KAN* |
| VL011 | *der1Δ* | MATa *his3 leu2 met15 ura3 der1Δ::KAN* |
| VL012 | *die2Δ* | MATa *his3 leu2 met15 ura3 die2Δ::KAN* |
| VL013 | *bst1Δ* | MATa *his3 leu2 met15 ura3 bst1Δ::KAN* |
| VL014 | *alg12Δ* | MATa *his3 leu2 met15 ura3 alg12Δ::KAN* |
| VL015 | *eug1Δ* | MATa *his3 leu2 met15 ura3 eug1Δ::KAN* |
| VL016 | *alg12Δire1Δ* | MATa *his3 leu2 met15 ura3 alg12Δ::KAN, ire1Δ::LEU2* |
| VL017 | *alg12Δhac1Δ* | MATa *his3 leu2 met15 ura3 alg12Δ::KAN, hac1Δ::LEU2* |
| VL018 | *bst1Δire1Δ* | MATa *his3 leu2 met15 ura3 bst1Δ::KAN, ire1Δ::LEU2* |
| VL019 | *bst1Δhac1Δ* | MATa *his3 leu2 met15 ura3 bst1Δ::KAN, hac1Δ::LEU2* |
| YMS101 | *ero1*-DAmP | MATa *his3 leu2 met15 ura3 ero1-DAmP::NAT* |
| YMS102 | *ero1*-DAmP | MATα *his3 leu2 lys2 ura3 ero1-DAmP::NAT* |
| BY4742 | WT | MATα *his3 leu2 lys2 ura3* |
| DC:126B11 | *alg3Δ* | MATα *his3 leu2 lys2 ura3 alg3Δ::KAN* |
| GS404 | *alg12Δ* | MATα *his3 leu2 lys2 ura3 alg12Δ::KAN* |
| DC:129E6 | *bst1Δ* | MATα *his3 leu2 lys2 ura3 bst1Δ::KAN* |
| DC:109G4 | *der1Δ* | MATα *his3 leu2 lys2 ura3 der1Δ::KAN* |
| DC:125C12 | *die2Δ* | MATα *his3 leu2 lys2 ura3 die2Δ::KAN* |
| DC:147F10 | *eug1Δ* | MATα *his3 leu2 lys2 ura3 eug1Δ::KAN* |
| GS984 | *fob1Δ* | MATα *his3 leu2 lys2 ura3 fob1Δ::KAN* |
| GS1295 | *hac1Δ* | MATα *his3 leu2 lys2 ura3 hac1Δ::KAN* |
| DC:107E2 | *hrd1Δ* | *MATα his3 leu2 lys2 ura3 hrd1Δ::KAN* |
| GS1293 | *ire1Δ* | *MATα his3 leu2 lys2 ura3 ire1Δ::KAN* |
| DC:119F6 | *mpd1Δ* | *MATα his3 leu2 lys2 ura3 mpd1Δ::KAN* |
| DC:106E9 | *ost3Δ* | *MATα his3 leu2 lys2 ura3 ost3Δ::KAN* |
| DC:103C11 | *ost6Δ* | *MATα his3 leu2 lys2 ura3 ost6Δ::KAN* |
| DC:103D4 | *sel1Δ* | *MATα his3 leu2 lys2 ura3 sel1Δ::KAN* |
| GS12 | SIR2OE | *MATα his3 leu2 lys2 ura3 SIR2OE::LEU2* |
| KS615 | SIR2OE*ire1Δ* | *MATα his3 leu2 lys2 ura3 SIR2OE::LEU2 ire1::KAN* |
| KS538 | SIR2OE*hac1Δ* | *MATα his3 leu2 lys2 ura3 SIR2OE::LEU2 hac1::KAN* |
| KS535 | *fob1Δhac1Δ* | *MATα his3 leu2 lys2 ura3 fob1::LEU2 hac1::KAN* |
| KS1651 | *tor1Δhac1Δ* | *MATα his3 leu2 lys2 ura3 tor1::URA3 hac1::KAN* |
