# Supplementary material for: Lifespan Extension Conferred by Endoplasmic Reticulum Secretory Pathway Deficiency Requires Induction of the Unfolded Protein Response
Source: PLoS Genet. 2014 Jan 2;10(1):e1004019. doi: 10.1371/journal.pgen.1004019 (PMC3879150; doi:10.1371/journal.pgen.1004019)
Supplement: Table S8 — Primers used for footprint and mRNA library preparation. (DOCX) [file pgen.1004019.s014.docx]

**Table S8. Primers used for footprint and mRNA library preparation.**

| **Name** | **Primer sequence** |
| --- | --- |
| Library_link1 | pCGTGATGATCGTCGGACTGTAGAACTCTGAACCTGTCGGTGGTCGCCGTATCATT/iSp18/CAAGCAGAAGACGGCATACGAATTGATGGTGCCTACAG |
| Library_link2 | pTGGTCAGATCGTCGGACTGTAGAACTCTGAACCTGTCGGTGGTCGCCGTATCATT/iSp18/CAAGCAGAAGACGGCATACGAATTGATGGTGCCTACAG |
| Library_link3 | pATTGGCGATCGTCGGACTGTAGAACTCTGAACCTGTCGGTGGTCGCCGTATCATT/iSp18/CAAGCAGAAGACGGCATACGAATTGATGGTGCCTACAG |
| Library_link4 | pCTGATCGATCGTCGGACTGTAGAACTCTGAACCTGTCGGTGGTCGCCGTATCATT/iSp18/CAAGCAGAAGACGGCATACGAATTGATGGTGCCTACAG |
| Library_link5 | pACATCGGATCGTCGGACTGTAGAACTCTGAACCTGTCGGTGGTCGCCGTATCATT/iSp18/CAAGCAGAAGACGGCATACGAATTGATGGTGCCTACAG |
| Library_link6 | pCACTGTGATCGTCGGACTGTAGAACTCTGAACCTGTCGGTGGTCGCCGTATCATT/iSp18/CAAGCAGAAGACGGCATACGAATTGATGGTGCCTACAG |
| Library_link7 | pGCCTAAGATCGTCGGACTGTAGAACTCTGAACCTGTCGGTGGTCGCCGTATCATT/iSp18/CAAGCAGAAGACGGCATACGAATTGATGGTGCCTACAG |
| Library_link8 | pTCAAGTGATCGTCGGACTGTAGAACTCTGAACCTGTCGGTGGTCGCCGTATCATT/iSp18/CAAGCAGAAGACGGCATACGAATTGATGGTGCCTACAG |
| Library_link9 | pGATCTGGATCGTCGGACTGTAGAACTCTGAACCTGTCGGTGGTCGCCGTATCATT/iSp18/CAAGCAGAAGACGGCATACGAATTGATGGTGCCTACAG |
| Library_link10 | pAAGCTAGATCGTCGGACTGTAGAACTCTGAACCTGTCGGTGGTCGCCGTATCATT/iSp18/CAAGCAGAAGACGGCATACGAATTGATGGTGCCTACAG |
| Library_link11 | pGTAGCCGATCGTCGGACTGTAGAACTCTGAACCTGTCGGTGGTCGCCGTATCATT/iSp18/CAAGCAGAAGACGGCATACGAATTGATGGTGCCTACAG |
| Library_link12 | pTACAAGGATCGTCGGACTGTAGAACTCTGAACCTGTCGGTGGTCGCCGTATCATT/iSp18/CAAGCAGAAGACGGCATACGAATTGATGGTGCCTACAG |
| bioAntiRiboPrime1-6 | 5`-bio\CAAGAGGTGCACAATCGACCGATCCTG;  5`-bio\ATATATTAGCATGGAATAATAGAATAGG;  5`-bio\CTACATGGTATAACTGTGGTAATTCTAG;  5`-bio\ATTAAGCCATGCATGTCTAAGTATAAGC;  5`-bio\TTGTTAATAGATAATATACGGATCTTAA;  5`-bio\TCGTCAATTAAAATGTTACGTGAGTTGGG |
| ill-cluster 3 | CAAGCAGAAGACGGCATACGA |
| ill-cluster 5 | AATGATACGGCGACCACCGA |

iSp18, internal spacer 18; p, phosphate; bio, biotin.
