# Supplementary material for: Lifespan Extension Conferred by Endoplasmic Reticulum Secretory Pathway Deficiency Requires Induction of the Unfolded Protein Response
Source: PLoS Genet. 2014 Jan 2;10(1):e1004019. doi: 10.1371/journal.pgen.1004019 (PMC3879150; doi:10.1371/journal.pgen.1004019)
Supplement: Text S1 — Supplementary text extending Materials and Methods. (DOCX) [file pgen.1004019.s015.docx]

**Supporting Information**

**Extended Materials and Methods**

**Ribosome profiling and mRNA sequencing**

Yeast cultures were grown to OD_600_ = 0.5 in 500 ml of complete YPD medium, and cells were collected by filtering through 0.45 μm filter (Millipore) with glass holder. Pellets were scraped with spatula, flash frozen in liquid nitrogen and stored at -80°C. To pharmacologically induce ER stress, tunicamycin was added into the medium at a final concentration 1 μg/ml, and cells were incubated at 30°C for an additional 30 min. Yeast extracts were prepared by cryogrinding the cell paste with BioSpec cryomill. Extracts were resuspended in 1 ml of ice-cold polysome lysis buffer [20 mM Tris-HCl (pH 8.0 at 4°C), 140 mM KCl, 5 mM MgCl_2_, 0.2 g/l cycloheximide, 1% triton-X100]. Ribosome fractionation, footprint library construction, and sequencing were performed as in [[1](#_ENREF_1)] with the following changes. Instead of polyadenylation, 3’ adapter (IDT, miRNA cloning linker #1) was attached to RNA footprint as an anchor for reverse transcription primer (Table S8). Linker ligation was set up overnight at 16°C in 10 μl reaction mixture: 3.75 μl water, 3 μl 50% PEG8000, 1 μl buffer (500 mM Tris-HCl, 10 mM MgCl_2_, 1 mM DTT, pH 7.5 at 25°C), 1 μl miRNA linker (100 ng/μl), 0.25 μl SuperaseIn (Invitrogen), 1 μl T4 ligase truncated K227Q (New England Biolabs). To remove contaminating ribosomal RNA, subtractive hybridization was performed using an antisense biotinylated DNA oligonucleotide mixture bioAntiRiboPrime1-6 (Table S8) attached to streptavidin dynabeads (Invitrogen) according to manufacturer’s protocol. An aliquot of cell lysates was used for total RNA extraction by hot acid phenol-chlorophorm. mRNA was isolated with poly(A) selection on oligo(dT) magnetic beads (mRNA DIRECT kit, Invitrogen). For RNA-seq, isolated mRNA was fragmented by alkaline solution [2 mM EDTA, 100 mM Na_2_CO_3_ (pH 9.2)], the fragments were resolved using a 15% TBE-urea gel, and the 50- to 75-nt region was cut from the gel. Further steps of library preparation were identical to those used for footprint libraries. Sequencing of footprint and RNA-seq libraries was performed on the Illumina HiSeq2000 platform. For preparation of each library barcoded RT-library 1-12 primers were used during reverse transcription step to allow multiplexing of samples for sequencing (Table S8).

**Analysis of heat and oxidative stress resistance**

Resistance of strains to heat stress was determined using spot assays. Cells were initially grown in liquid culture until OD_600_ = 0.6, and 10x serial dilutions for each strain were spotted on YPD solid medium. The plates were incubated at either 30° or 40°C, and images were taken 48 h after plating. For measuring oxidative stress resistance, strains were grown in YPD until OD_600_ = 0.6 and treated with 1 mM hydrogen peroxide for 30 min. Cells were centrifuged, washed with YPD medium and serial dilutions were plated on YPD agar plates. Colony numbers were counted 48 h after plating.

**References**

1. Gerashchenko MV, Lobanov AV, Gladyshev VN (2012) Genome-wide ribosome profiling reveals complex translational regulation in response to oxidative stress. Proc Natl Acad Sci U S A 109: 17394-17399.
